# Association between nutrients and metabolic syndrome in middle-aged Korean women

**DOI:** 10.20945/2359-3997000000252

**Published:** 2020-06-05

**Authors:** Sul Lee, Hyun Joo Lee, Seung Chul Kim, Jong Kil Joo

**Affiliations:** 1 Pusan National University School of Medicine Pusan National University Hospital Busan Korea Department of Obstetrics and Gynecology, Pusan National University School of Medicine, Pusan National University Hospital Medical Research Institute, Busan, Korea

**Keywords:** Metabolic syndrome, nutrients, dietary pattern, carbohydrate, protein

## Abstract

**Objectives:**

The aim of this study was to evaluate the association between nutritional intake and metabolic syndrome in otherwise healthy middle-aged Korean women.

**Subjects and methods:**

Retrospectively, medical records were reviewed for nutritional intake of 2,182 Korean women who had undergone routine medical check-ups from 2010 to 2016 at Pusan National University Hospital. The patients who met diagnostic criteria for metabolic syndrome based on NCEP-ATPIII were included, and each of the patients was assessed through self-report questionnaires and individual interview with a health care provider. The recommended dietary allowance (RDA) for women in Republic of Korea was based on 2015 criteria discussed in Dietary Reference Intake for Koreans, organized by the Ministry of Health and Welfare.

**Results:**

Through univariate analysis, daily calorie, protein, fat, and carbohydrate consumption were significantly higher and exceeded RDA in the patients with metabolic syndrome; other than major nutrients, iron, vitamin B2, and niacin were also consumed in excess of the RDA in these patients. Multivariate analysis showed that carbohydrate consumption, along with protein and vitamin B2, were significantly higher in the patients with metabolic syndrome.

**Conclusion:**

In middle-aged Korean women, high consumption of carbohydrates, along with protein and vitamin B2, was found to have a statistically significant association with the presence of metabolic syndrome. Arch Endocrinol Metab. 2020;64(3):298-305

## INTRODUCTION

Metabolic syndrome (MetS), one of the largest-growing health concerns worldwide, is a cluster of diseases based on physiological consequences of insulin resistance including obesity, hypertension, diabetes mellitus, and dyslipidemia ( 1 ). As a degenerative disorder that worsens with age, its close relation to cardiovascular risks along with increased morbidity and mortality is widely known, and patients require education and lifelong management ( 2 ).

One of the major issues in public education for MetS patients is diet; dietary patterns and nutrients intake likely determine the development and/or progress of the disease, even seemingly accelerating its prevalence ( 2 - 6 ). However, it is not clear exactly which nutrients affect MetS. Several nutritional factors have been reported to be associated with MetS, but the results are not consistent ( 5 , 7 , 8 ).

For such reasons, numerous national, comprehensive studies on dietary patterns and nutrients in MetS patients are now being conducted worldwide; in the United States and Canada, comparative studies on dietary patterns and nutrients intake of general population and patients with MetS have found parallel associations between higher intake of saturated fatty acids – saturated foods and soft drinks, for examples – and higher risk for MetS ( 9 , 10 ). Similarly, research in East Asian countries including China and Japan has shown that a high-protein/cholesterol diet is associated with higher prevalence of MetS; researchers have emphasized the importance of developing nutritional intervention for not only management but also preventive purposes ( 11 - 13 ).

In South Korea, the prevalence of MetS is increasing, as it is in other Asian countries. From 1998 to 2012, the prevalence rose from 24.9% to 37% ( 3 , 14 ). Over the past several decades, eating habits in Korea have become westernized (high intakes of beef, sugar, and fat) and can be easily ingested through a variety of automated processes. In this process, a meat diet preference became more favored than vegetarian diets, and carbohydrate consumption increased ( 15 ).

Therefore, in this study, we attempted to determine how individuals with metabolic syndrome take nutrients and how it differs from how individuals without metabolic syndrome do so.

## SUBJECTS AND METHODS

### Study cohort and design

This was a retrospective study of 2,182 women who underwent health screening examinations at the Pusan National University Hospital Health Promotion Center in South Korea between 2010 and 2016. This period, about 4,000 female patients visited the Health Promotion Center of Pusan National University Hospital, of which 2,200 middle-aged women (aged 30-74) were selected. When they first visited, we offered a self-administered questionnaire including each participant’s demographics, lifestyle, medical history, and diet. All the participants agreed with their informed consent that their medical records would be used in this study. After completing the questionnaire, the nutritionist checked again through individual interviews, so few surveys were missing, and missing FFQs were excluded from the data. Therefore, a total of 2,182 people were selected, except for 18 surveys of some inaccurate FFQs. We divided them into patients with and without MetS: 1,805 women without MetS and 377 women with MetS. This study was approved by the Institutional Review Board (IRB) of Pusan National University Hospital (H-1906-023-080).

### Definition of metabolic syndrome

We used the National Cholesterol Education Program’s Adult Treatment Panel III report to define MetS (16); women needed to meet at least three of the following five criteria; ( 1 ) abdominal circumference over 80 cm (for Asian women), ( 2 ) TG level over 150 mg/dL, ( 3 ) HDL-C less than 50 mg/dL, ( 4 ) FBG over 110 mg/dL or DM, and ( 5 ) blood pressure over 130/85 or hypertension medication ( 17 ).

### Nutrition Intake survey

We conducted the nutrition survey through direct interviews with patients who visited the Pusan National University Hospital Health Promotion Center. The questionnaire items were prepared based on the revised edition of Health Promotion of Koreans in 2004. We calculated the nutrition data for each food using a semi-quantitative food frequency survey.

The FFQ we used was based on Korean National Health and Nutrition Survey form. This FFQ is a format commonly used in other nutrient articles ( 18 ). It consists of 32 questions and results were expressed as a sum of weekly food consumption based on 1 serving size. 32 questions were composed of grains, meat and fishes, vegetables, fats, milk products, fruits, sugars, and others. Grains include bread, sponge cake, cake, crackers, biscuits, cooked rice, noodle, rice, potato, etc. Meat and fishes include beef, pork, chicken, low fat fishes, squid, shrimp, crab, shellfish, oyster, anchovy, dried white bait, eggs, medium fat fishes, tofu, black bean, hams, high fat fishes, rib, sausage, eel. Vegetables include green-yellow, white, young radish, radish leaves, kimchi, diced radish kimchi, seaweeds. Fats include nuts, plant oils, mayonnaise, butter, creams, bacon. Milk products include milk, yoghurt, ice cream. Fruits include fresh fruit, fresh juice. Others include sugars (sugar/syrup/sweetened drink/candy/chocolate) and others (fast-foods, instant noodle, Korean stews, coffee/tea). The daily recommended dietary allowances of Koreans were based on 2015 criteria for Dietary Reference Intake for Koreans (2015 KDRIs) organized by the Ministry of Health and Welfare. We divided each nutrient into the actual daily intake and the percentage of actual intake compared with daily recommended nutrients intake (RNI) in percentage.

Also, nutrients analysis used at Pusan National University Hospital software is a program developed by itself, but this program is based on CAN-pro 3.0 made by the Korean Nutrition Society. This program is widely used in Korea ( 19 , 20 ).

### Statistical analysis

In this study, we used SAS 9.3 for statistical analysis, the independent t test or Wilcoxon rank-sum test for continuous variables and the chi-square test for categorical variables. To calculate the area under the receiver operating characteristic curve, we used a logistic regression model. We considered two-sided values of P < 0.05 statistically significant.

## RESULTS

### Demographics and characteristics

Characteristics of the patients both with and without MetS are shown in Table 1 . We found statistically significant differences in most parameters but not the following: smoking, total bilirubin, direct bilirubin, albumin, creatinine, kcal, protein (g), calcium (mg), calcium (percent), vitamin A (Retinol Equivalent, RE), vitamin A (percent), vitamin B2 (mg), vitamin C (mg), vitamin C (percent). With the physical measurements, all parameters were higher in patients with MetS. The syndrome was more commonly observed in menopausal women as well as in women with diabetes mellitus, high blood pressure, and dyslipidemia.

**Table 1:** Demography and characteristics

Variables	Total (n = 2,182)	Without MetS (n = 1,805)	With MetS (n = 377)	*p
**Clinical characteristics**				
Age (yr)*	53.09 (10.00)	51.68 (9.69)	59.86 (8.62)	<.0001
Body weight (kg)*	56.77 (7.89)	55.6 (7.01)	62.38 (9.33)	<.0001
Percent body fat (%)**	29.20 [26.00, 32.30]	28.40 [25.30, 31.30]	33.10 [30.90, 35.50]	<.0001
WC (cm)*	78.76 (8.46)	77.07 (7.49)	86.88 (8.13)	<.0001
BMI (kg/m^2^)*	22.64 (3.03)	22.07 (2.58)	25.36 (3.54)	<.0001
Height (cm)*	158.40 (5.26)	158.72 (5.10)	156.86 (5.74)	<.0001
Body muscle (kg)*	36.73 (3.51)	36.50 (3.27)	37.83 (4.34)	<.0001
Skeletal muscle (kg)*	18.52 (3.81)	18.25 (3.69)	19.80 (4.10)	<.0001
**Menopause (%)**				<.0001
Pre-menopause	818 (37.49)	762 (42.22)	56 (14.85)	
Post-menopause	1364 (62.51)	1043 (57.78)	321 (85.15)	
**Hypertension (%)**				<.0001
without hypertension	1803 (82.63)	1635 (90.58)	168 (44.56)	
with hypertension	379 (17.37)	170 (9.42)	209 (55.44)	
**DM (%)**				<.0001
without DM	2092 (95.88)	1781 (98.67)	311 (82.49)	
with DM	90 (4.12)	24 (1.33)	66 (17.51)	
**Hyperlipidemia (%)**				<.0001
Without Hyperlipidemia	1841 (84.37)	1617 (89.58)	224 (59.42)	
With Hyperlipidemia	341 (15.63)	188 (10.42)	153 (40.58)	
**Smoking (%)**				0.300
Non-smoking	2072 (94.96)	1710 (94.74)	362 (96.02)	
Smoking	110 (5.04)	95 (5.26)	15.00 (3.98)	
**Group (%)**				<.0001
Premenopause, no MetS	762 (34.92)	762 (42.22)	0 (0.00)	
Premenopause, MetS	56 (2.57)	0 (0.00)	56 (14.85)	
Postmenopause, no MetS	1043 (47.80)	1043 (57.78)	0 (0.00)	
Postmenopause, MetS	321 (14.71)	0 (0.00)	321 (85.15)	
**Laboratory parameters**				
LDH (IU/L)*	270.12 (95.77)	265.91 (94.03)	290.29 (101.45)	<.0001
Total_Bilirubin (mg/dL)*	0.87 (0.37)	0.87 (0.37)	0.85 (0.36)	0.239
Direct_Bilirubin (mg/dL)*	0.22 (0.09)	0.22 (0.09)	0.21 (0.08)	0.051
Total_Protein (g/dL)*	7.16 (0.41)	7.15 (0.41)	7.23 (0.40)	<.0001
Albumin (g/dL)*	4.41 (0.26)	4.41 (0.26)	4.42 (0.26)	0.608
BUN (mg/dL)**	13.50 [11.20, 16.00]	13.40 [11.20, 15.90]	14.10 [11.30, 16.70]	0.008
Creatinine (mg/dL)**	0.69 [0.63, 0.77]	0.69 [0.63, 0.77]	0.70 [0.62, 0.79]	0.590
Calculated GFR (mL/min)*	90.21 (17.84)	90.85 (17.25)	87.16 (20.16)	0.001
r_GTP (IU/L)**	16.00 [12.00, 23.00]	15.00 [12.00, 21.00]	22.00 [16.00, 32.00]	<.0001
Calcium (mg/dL)*	9.41 (0.38)	9.39 (0.38)	9.48 (0.38)	<.0001
ALP (IU/L)**	66.00 [52.00, 91.00]	63.00 [50.00, 87.00]	75.00 [60.00, 116.00]	<.0001
Total_Cholesterol (mg/dL)*	201.00 (37.00)	199.37 (34.88)	208.82 (45.06)	<.0001
Triglyceride (mg/dL)**	81.00 [59.00, 113.00]	74.00 [56.00, 100.00]	129.00 [94.00, 177.00]	<.0001
HDL_Cholesterol (mg/dL)*	62.28 (15.45)	64.93 (14.76)	49.6 (12.01)	<.0001
LDL_Cholesterol (mg/dL)*	127.09 (35.28)	124.55 (33.1)	139.26 (42.24)	<.0001
Free_Fatty_Acid (uEq/L)**	598.00 [434.00, 803.00]	585.00 [425.00, 794.00]	651.00 [466.00, 842.00]	0.001
Glucose (mg/dL)**	86.00 [81.00, 93.00]	85.00 [80.00, 91.00]	97.00 [88.00, 108.00]	<.0001
Insulin_RIA (uIU/mL)**	4.17 [3.37, 5.18]	4.01 [3.29, 4.90]	5.13 [4.20, 7.53]	<.0001
CRP (mg/dL)**	0.04 [0.02, 0.09]	0.03 [0.02, 0.07]	0.08 [0.04, 0.15]	<.0001
**IDMS_MDRD_ckd (%)**				<.0001
Without CKD	2130 (97.62)	1780 (98.61)	350 (92.84)	
With CKD	52 (2.38)	25 (1.39)	27 (7.16)	
**Nutritional data (derived from the FFQ)**				
Kcal*	1754.13 (306.17)	1751.94 (297.22)	1764.61 (346.08)	0.509
Kcal_percent**	101.76 [88.48, 118.04]	99.50 [87.21, 113.98]	115.82 [97.54, 130.52]	<.0001
Protein_g*	68.00 (14.81)	67.99 (14.49)	68.07 (16.26)	0.932
Protein_percent*	106.80 (27.65)	104.44 (26.98)	118.12 (28.05)	<.0001
Fat_g*	44.86 (12.96)	45.28 (12.78)	42.89 (13.62)	0.001
Fat_percent*	118.53 (36.7)	117.21 (36.11)	124.87 (38.83)	<.0001
Carbohydrate_g**	269.50 [235.50, 302.30]	268.30 [235.75, 300.30]	274.95 [235.30, 316.45]	0.007
Carbohydrate_percent**	95.42 [81.58, 113.26]	93.16 [80.42, 108.22]	112.05 [91.13, 128.70]	<.0001
Calcium_mg*	898.08 (243.61)	893.78 (243.40)	918.68 (243.86)	0.071
Calcium_percent*	128.30 (34.80)	127.68 (34.77)	131.24 (34.84)	0.071
Iron_mg*	19.32 (4.61)	19.23 (4.55)	19.75 (4.84)	0.048
Iron_percent*	146.58 (41.90)	143.96 (41.50)	159.13 (41.59)	<.0001
VitA_RE*	1091.58 (323.16)	1086.81 (322.73)	1114.39 (324.66)	0.132
VitA_percent*	155.75 (46.21)	155.03 (46.15)	159.20 (46.38)	0.112
VitB1_mg*	1.14 (0.23)	1.14 (0.23)	1.19 (0.25)	<.0001
Vit B1_percent**	86.15 [75.38, 98.46]	85.50 [74.62, 96.92]	90.77 [76.92, 103.08]	<.0001
VitB2_mg*	1.49 (0.40)	1.49 (0.40)	1.52 (0.40)	0.148
Vit B2_percent*	98.48 (26.71)	97.90 (26.63)	101.27 (26.94)	0.026
Niacin_mg*	17.44 (3.42)	17.32 (3.33)	17.99 (3.77)	0.002
Niacin_percent*	122.52 (24.55)	121.34 (23.93)	128.15 (26.66)	<.0001
VitC_mg*	79.49 (24.79)	79.12 (24.66)	81.27 (25.38)	0.126
vitC_percent*	113.2 (35.87)	112.65 (35.69)	115.85 (36.66)	0.115
Alcohol_kcal**	0.00 [0.00, 14.29]	0.00 [0.00, 14.29]	0.00 [0.00, 0.00]	0.003

Data are presented as means. *, mean(SD); **, mean[IQR]; percent, daily intake/daily recommendation intake quantity × 100.

p is by independent t test.

MetS: metabolic syndrome; WC: waist circumference; BMI: body mass index; DM: diabetes mellitus; LDH: lactate dehydrogenase; BUN: blood urea nitrogen; MDRD: modification of diet in renal disease; IDMS: isotope dilution mass spectrometry; γ-GTP: gamma-glutamyltransferase; ALP: alkaline phosphatase; HDL: high density lipoprotein; LDL: low density lipoprotein; RIA: radioimmunoassay; CRP: C-reactive protein; Kcal, kilocalories; Vit: Vitamin.

### Relationship MetS and nutrition

In terms of nutrients intake, the percentage of total calories in the women with metabolic syndrome was higher and the intake of fat, protein, carbohydrate, iron, vitamin B1 and niacin was also higher. In univariate analysis, the intake of energy-related factors such as caloric intake, protein, fat and sugar was statistically significantly higher than the recommended daily allowance (RDA) in MetS patients. Iron (mg) and vitamin B2 (mg) in daily total intake quantity and niacin (mg and percent) in both daily total intake quantity and percentage of the total daily intake compared with the RDA also showed statistically significant results. However, calcium(mg and percent), vitamin A (mg and percent), and vitamin C (mg and percent) in both daily total intake quantity and percentage of the total daily intake compared with the RDA and alcohol intake (kcal) in daily total intake, vitamin B1 (percent) in daily recommended did not produce statistically significant results ( Table 2 ). By multivariate analysis, only protein(percent), carbohydrate (percent), and vitamin B2 (percent) in percentage of the total daily intake compared with the RDA were statistically significantly higher in patients with MetS ( Table 3 ).

**Table 2:** Logistic regression (univariate) analysis of nutrition and metabolic syndrome

Response	Predictors	OR	95% CI		p-value

			Lower	Upper	
Mets	Kcal_kcal	1.000	1.000	1.000	0.465
	Kcal_percent	1.021	1.016	1.026	<.0001
	Protein_g	1.000	0.993	1.008	0.926
	Protein_percent	1.018	1.014	1.022	<.0001
	Fat_g	0.986	0.977	0.994	0.001
	Fat_percent	1.006	1.003	1.009	<.0001
	Carbohydrate_g	1.003	1.001	1.005	0.003
	Carbohydrate_percent	1.014	1.010	1.019	<.0001
	Calcium_mg	1.000	1.000	1.001	0.071
	Calcium_percent	1.003	1.000	1.006	0.071
	Iron_mg	1.024	1.000	1.049	0.048
	Iron_percent	1.008	1.006	1.011	<.0001
	VitA_RE	1.000	1.000	1.001	0.132
	vitA_percent	1.002	1.000	1.004	0.112
	VitB1_mg	2.497	1.547	4.031	<.0001
	Vit B1_percent	1.002	0.999	1.004	0.165
	VitB2_mg	1.223	0.931	1.607	0.148
	Vit B2_percent	1.005	1.001	1.009	0.026
	Niacin_mg	1.057	1.024	1.092	0.001
	Niacin_percent	1.011	1.007	1.016	<.0001
	VitC_mg	1.003	0.999	1.008	0.126
	vitC_percent	1.002	0.999	1.006	0.115
	Alcohol_kcal	1.000	0.999	1.001	0.773

MetS: metabolic syndrome; Kcal: kilocalories; Vit: vitamin; OR: odds ratio, percent, daily intake/daily recommendation intake quantity × 100.

**Table 3:** Logistic regression(multivariate) analysis of nutrition and metabolic syndrome

Response	Predictors	OR	95% CI		p-value

			Lower	Upper	
Mets	Age	1.095	1.078	1.112	<.0001
	Kcal_percent	1.005	1.000	1.010	0.065
	Protein_percent	1.011	1.004	1.017	0.001
	Carbohydrate_percent	1.003	1.000	1.006	0.026
	Iron_percent	0.998	0.991	1.005	0.586
	VitB2_percent	0.996	0.987	1.005	0.391
	Niacin_percent	1.000	0.992	1.007	0.957

MetS: metabolic syndrome; Kcal: kilocalories; Vit: vitamin; OR: odds ratio; percent, daily intake/daily recommendation intake quantity × 100.

## DISCUSSION

Because MetS is a degenerative disease, women with MetS are older, as shown in Table 1 . Likewise, factors that reflect lifestyle or diet habits such as BMI, WC, and body weight were also higher in women with MetS. In univariate analysis, carbohydrate, protein, fat, iron, vitamin A, vitamin B2, and niacin were more positively correlated with MetS. However, on multivariate analysis, only protein and carbohydrate were associated with MetS in percentage of the total daily intake compared with the RDA.

Authors of several studies have reported the association between protein intake and incidence of MetS. In a study on Koreans, being male had a positive correlation with MetS when the diet was high in meat (multivariate-adjusted PR of the highest group compared with the lowest group: 2.15, 95% CI: 1.10-4.21, p for trend = 0.005). However, women’s dietary patterns were not related to MetS ( 3 ). In Chinese adults, higher scores for the high-protein/cholesterol pattern were associated with higher prevalence of MetS (OR for the extreme quartile: 1.36, 95% CI, 1.10-1.68, p for trend < 0.01). High-protein/cholesterol pattern includes high intakes of animal offal, animal blood, and sausage ( 11 ). Most of the researchers who analyzed the dietary patterns of people with MetS found an association with high protein intake. In this study, however, we found that women with MetS took in more protein than the recommended daily protein intake but no connection with total amount of protein intake.

In 2015 KDRI, the appropriate energy ratio is 55%-65% for carbohydrates. In our data, total/without MetS/with MetS was 61.45%/61.25%/62.32% for carbohydrates, respectively. Our data did not exceed AMDR in all groups, but it is higher intake of carbohydrate in women with MetS compared to other groups. Korean foods have a higher proportion of carbohydrates than Western foods, and in Korea, the proportions of energy from carbohydrates, 26.0% for men and 25.2% for women, were in the recommended range, whereas 58.0% of men and 60.0% of women exceeded AMDR from carbohydrates (65%) ( 21 ). Higher carbohydrate intake (> 80% of energy from total intake quantity) is associated with MetS in men, but the relationship is not statistically significant in women ( 21 ). According to a paper based on the 2005 Korean National Health and Nutrition Examination Survey, the traditional Korean diet (high carbohydrate and low animal fat with ample plant foods) may affect the incidence of MetS ( 22 ).

High carbohydrate consumption may be related with insulin resistance; people with pathological insulin resistance have a higher risk of developing MetS and are more likely to progress to DM ( 23 ). Women with insulin resistance had significantly higher total carbohydrate intake and energy from carbohydrates than did women who did not have insulin resistance ( 24 ). Other studies have shown that excessive carbohydrate intake can comprise a relatively low-fat diet, which in turn can reduce high-density lipoprotein cholesterol ( 25 ). Also, consumption of refined carbohydrates, such as white rice, can cause increased abdominal circumference ( 26 ). All these results suggest high carbohydrate consumption as a risk factor for development of MetS. Our study also showed that increased carbohydrate intake in women was associated with increased prevalence of MetS.

Metabolic syndrome is also associated with energy-related nutrients such as proteins and carbohydrates, as well as co-factors that affect metabolism such as vitamins and minerals. Vitamin B2 (riboflavin) is known to be essential for coenzyme synthesis for glucose, fatty acids, glycerol, and amino acid for energy ( 27 ). In a mouse experiment, riboflavin deficiency causes adipocyte lipolysis and apoptosis, leading consequently to the severe chronic inflammation that accompanies obesity ( 28 ). Research on humans has not yet produced results. In our study, univariate analysis showed that over-intake of vitamin B2 was positively related to MetS, but the relationship was not statistically significant in multivariate analysis.

Vitamin B3 (niacin) is known to increase the levels of HDL cholesterol and reduce triglyceride and LDL cholesterol levels. However, it is reported that diabetic patients may exacerbate their blood glucose control status ( 29 ). Niacin, also known as methyl-consumer, is toxic through various mechanisms including glucose intolerance, insulin resistance, elevated liver enzymes, fatty liver, and steatosis ( 30 ). In 2010, Li and cols. reported a strong lag-correlation between obesity, diabetes prevalence, and capita consumption of niacin in the United States ( 31 ). In our study, on univariate analysis, vitamin B3 was associated with MetS but not on multivariate analysis.

The limitation of our study is that the target sample was relatively small and was based on data from the limited areas of Busan and Gyeongnam of South Korea. We believed that there would be differences in survey methods based on questionnaire preparation because the absolute amount of actual nutrient intake may vary. However, this was a relatively uniform sample because the women we studied likely used similar ingredients and relatively similar recipes in a limited area. Second, there was no analysis of several factors that induce MetS: fat composition, physical activity, and food quality. Third, the relationship between MetS and Menopause was also investigated in Korean middle-aged women, but no analysis was conducted between the two. Next time, this will be followed by a new study analyzing nutrients, MetS and menopause. Fourth, because our data are based only on RNI for each nutrient, there is a lack of adequate assessment for groups above or below RNI in all groups.

In conclusion, MetS is a disease that presents as various diagnoses, and it is probably right to aim at correcting living and dietary habits that lead to MetS rather than for therapeutic purposes themselves. Koreans consume the most carbohydrates in Asian countries, where rice is the staple food. In addition, these carbohydrates mostly are included foods with high glycemic index, such as refined rice, bread, ramen, and noodles. As animal proteins such as pork, chicken, and beef are readily available, protein intake from Koreans also exceeds the recommended amount for most age groups. In Koreans, excessive intake of carbohydrates and proteins is also a problem, but high intake of purified carbohydrates and animal proteins can be more problematic. In our study, the dietary habits of high carbohydrate and high protein were related to MetS, therefore, it will be necessary to convert the intake of carbohydrates and proteins to an appropriate amount of energy and convert to less purified carbohydrates and protein such as soy and fish.
